# Rhizosphere-colonizing bacteria persist in the protist microbiome

**DOI:** 10.1128/msphere.00037-25

**Published:** 2025-04-30

**Authors:** Stephen J. Taerum, Ravikumar R. Patel, Justin E. Alamo, Daniel Gage, Blaire Steven, Lindsay R. Triplett

**Affiliations:** 1 Department of Plant Pathology and Ecology, The Connecticut Agricultural Experiment Station, New Haven, Connecticut, USA; 2 Department of Molecular and Cell Biology, The University of Connecticut, Storrs, Connecticut, USA; 3 Department of Environmental Science and Forestry, The Connecticut Agricultural Experiment Station, New Haven, Connecticut, USA; Ben-Gurion University of the Negev, Beer-Sheva, Israel

**Keywords:** protist, rhizosphere, Colpoda, microbiome, maize

## Abstract

Soils contain diverse predatory protists that affect the abundance and behavior of rhizosphere bacteria, including bacteria that may benefit plant health. Protists harbor their own bacterial microbiomes, and we previously observed that plants inoculated with protists harbored rhizosphere bacteria similar to those in the protist inoculum. To determine how protist microbiomes affect the rhizosphere, we profiled the bacteria of eight diverse rhizosphere protist isolates after 2 years of laboratory culture. We then compared the protist culture microbiomes to maize rhizosphere communities 6 weeks after protist inoculation. Introduction of protists enriched 13 protist-associated bacterial amplicon sequence variants (ASVs) in the rhizosphere, which comprised ~10% of the rhizosphere bacterial community. Additional bacterial ASVs ranked highly in abundance in both rhizosphere (top 100) and protist (top 20) microbiomes; together, a median 47% of the protist microbiome was enriched or in high rank abundance in the rhizosphere. Inoculation with three out of eight protist cultures positively affected root biomass traits, but a protist mixture had no effect, indicating that the impact of protist-associated bacteria on plant growth is context dependent. Isolates of protist-associated bacteria had both positive and negative effects on protist growth in culture, suggesting that the bacteria use multiple strategies to survive in proximity to predators. This study demonstrates that even after long-term laboratory culture, rhizosphere protist cultures host bacteria that can colonize the rhizosphere of maize. The findings also identify diverse groups of rhizosphere-colonizing bacteria that persist among protist predators, which suggests that these bacteria could associate with or benefit from protists in the soil.

**IMPORTANCE:**

Understanding the impact of predatory protists on the plant microbiome will be essential to deploy protists in sustainable agriculture. This study shows that eight rhizosphere protist isolates hosted diverse and distinct bacterial communities and that a large proportion of these bacteria could be found colonizing the maize root environment 6 weeks after protists were inoculated onto seedlings. This study demonstrates that certain bacteria from the maize rhizosphere can persist for years in protist cultures and retain the ability to colonize rhizosphere soil, suggesting that protists might support the survival of these rhizosphere bacteria in the absence of the plant.

## INTRODUCTION

Microbial eukaryotes including fungi, nematodes, and protists are integral to the plant microbiome. Single-celled protists—eukaryotes that cannot be classified as plant, animal, or fungus—are ubiquitous in soil and are often predators of bacteria and fungi (1). Protists may stimulate plant growth, and this was historically often attributed to the stimulation of nutrient cycling (2). There is growing consensus that predators provide benefits to plant growth and disease resilience through selective predation. Culling prey bacteria can increase the competitive fitness of predation-resistant bacteria, including plant growth-promoting strains of *Bacillus*, *Sphingomonas*, *Pseudomonas*, and *Azospirillum* (3). Although few species of rhizosphere bacteria are reported to derive fitness benefits from protists, network association studies have identified hundreds of positive correlations among protist and bacterial groups in the rhizosphere (4–6). This suggests that a much wider diversity of rhizosphere bacteria may have positive ecological associations with predators than is currently established. Given that correlations could arise from niche constraints, environmental factors, or random chance (7), systematic studies are needed to understand which rhizosphere-colonizing bacteria are positively selected by protists.

Rhizosphere eukaryotes interact with bacteria, but they also host their own bacterial microbiomes that shape their interaction with plants. For example, nematode endosymbionts detoxify plant secondary metabolites; endofungal *Rhizobium* and *Burkholderia* spp. promote plant-beneficial fungi and pathogenic fungi; and *Fusarium*-colonizing *Pantoea* spp. inhibit virulence by producing antifungal compounds (8–11). Highly cosmopolitan multi-kingdom colonists, including some strains of *Bacillus* and *Pseudomonas* bacteria, may even be transferred from fungi or nematodes to plants (12, 13). Protists host endosymbiotic, transient intracellular, and extracellular bacterial communities through a wide range of mutualistic and antagonistic mechanisms (14). The model amoeba *Dictyostelium discoideum* hosts both prey and predation-resistant bacteria, which may be obligate or transient residents (15). Green algae microbiomes parallel those of plant roots and include many bacteria that can colonize and benefit both organisms (16). No studies to our knowledge have characterized bacteria associated with other groups of rhizosphere protists, but an understanding of such communities could point to synergisms that benefit the plant. For example, nitrogen-fixing *Sinorhizobium meliloti* is a transient resident of ciliate cells, and association with protists increases the bacteria’s distribution and nodulation capacity (17, 18).

We previously observed that after inoculating plants with a defined protist consortium, the rhizosphere community contained many bacterial taxa found in the protist cultures that were used to inoculate the plants (19). Protist inoculation also alleviated a negative growth effect of field soil bacteria, and this impact was dependent on bacteria in the protist culture. These findings led us to hypothesize that protist-associated bacteria (i.e., the bacterial microbiome of the protist) could help establish a beneficial rhizosphere microbiome. In this study, we first identified bacterial taxa found in long-term culture with rhizosphere protist isolates. Although the nature of their interaction with protists is not known, we term these “protist-associated bacteria” for brevity. Second, we tracked the abundance of those bacterial taxa after protist inoculation onto plants. We asked:

- Are protist-associated bacteria enriched in the rhizosphere after protist inoculation?
- Do different protist cultures have different effects on the rhizosphere microbiome?
- Is bacterial establishment in the rhizosphere affected when protists are inoculated individually or in a mixture?
- Finally, how do protist-associated bacteria affect protist growth?

We find that bacterial taxa associated with evolutionarily diverse protists can colonize the rhizosphere, suggesting that protists could affect rhizosphere health through shared microbiota.

## RESULTS

### Characterization of bacteria associated with rhizosphere protist isolates

Protists were previously isolated from independent maize root samples at two sites (20) and maintained in laboratory culture for over 2 years without the addition of live bacteria. Eight protist isolates were selected for characterization of their bacterial microbiome. Five of the selected protists are known to colonize the maize rhizosphere after inoculation to roots (*Colpoda*, *Cercomonas*, *Ochromonas*, *Thaumatomonas*, and Chrysophyceae sp.; Table S1) (19). Three isolates were selected to represent the groups Amoebozoa and Rhizaria (*Flamella* and *Nuclearia* spp.) and *Allapsa*, a genus with significance to plant health (5). Sequence profiling of 16S rRNA genes in twice-washed protist cultures identified 30–75 bacterial genera in each protist microbiome (Table S1). Protist-associated bacterial communities had lower Shannon diversity indices than bacteria cultured from bulk and rhizosphere soils collected at the original protist isolation sites, which we sequenced concurrently and used as bacterial inoculum for plants (Table S1).

**Fig 1:**
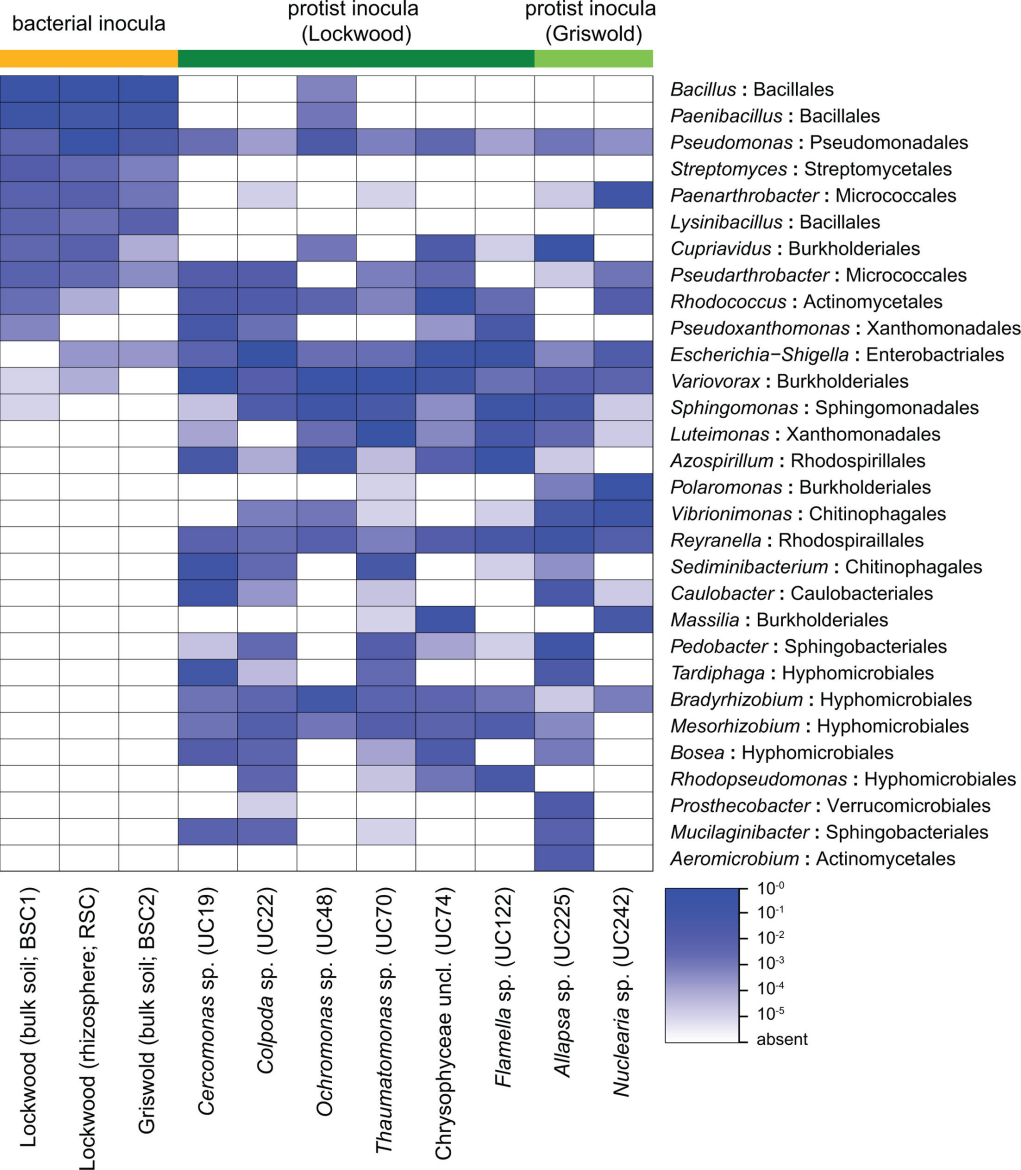
Bacterial microbiome composition of the protist-free bacterial inocula and the eight protist isolates from the maize rhizosphere. Only the “dominant genera,” i.e., the genera having the 30 highest mean abundances across samples, are shown. Bacterial order is indicated after the colon. The shade corresponds to proportional abundance in the culture sample.

We identified the 30 most abundant bacterial genera, belonging to 15 orders, across protist and soil samples (henceforth referred to as “dominant genera”) (Fig. 1). While each microbiome was distinct, there were five core dominant genera detected among all protist-associated bacterial communities (*Pseudomonas*, *Variovorax*, *Sphingomonas*, *Reyranella*, and *Bradyrhizobium*) and eight additional genera represented in six of eight communities. However, there were no core amplicon sequence variants (ASVs) of *Sphingomonas* or *Reyranella* (Table S2), showing that although these genera were ubiquitous among cultures, they were represented by different ASVs across the different protist cultures. Members of Burkholderiales and Hyphomicrobiales were ubiquitously abundant in protist-associated bacterial communities; nine of the dominant bacterial genera represented these two orders. The results indicate that while each protist culture enriched a distinct subset of bacteria after isolation from rhizosphere soil, certain bacterial taxa broadly persisted in association with diverse rhizosphere protists.

### Establishment of protists and protist-associated bacteria in the rhizosphere

**Fig 2:**
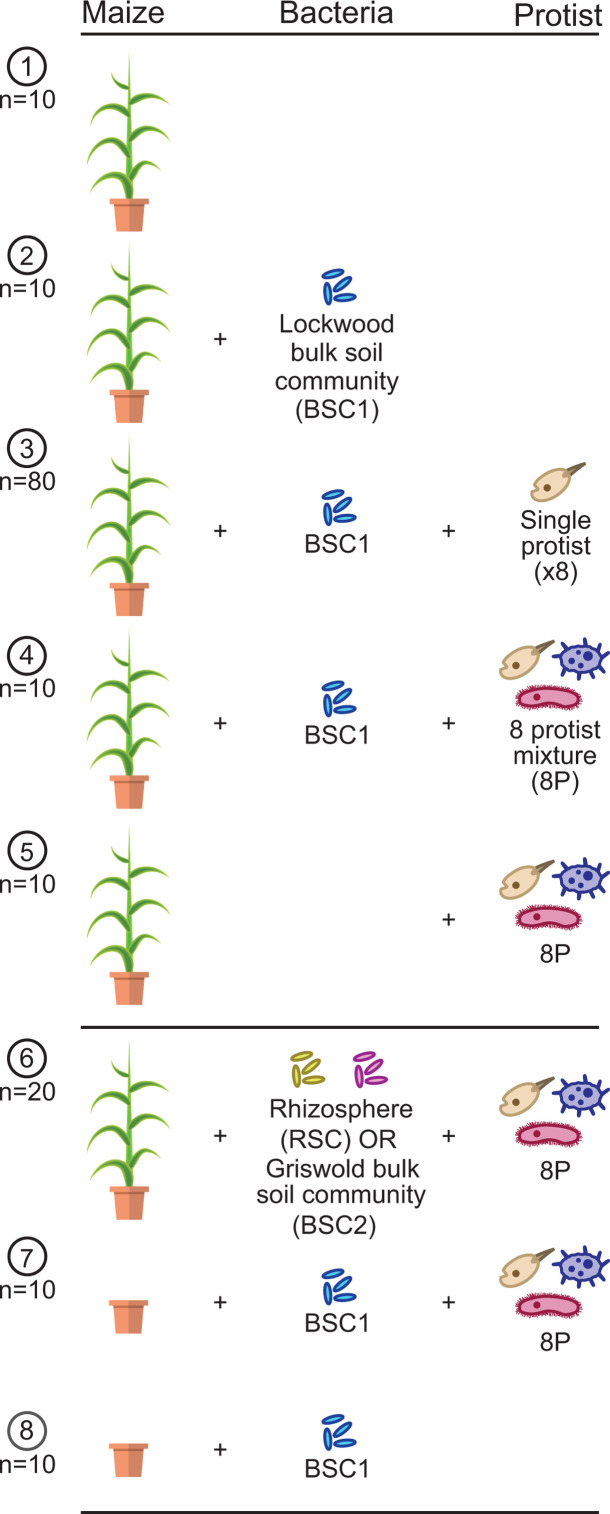
Experimental design showing eight combinations of protist and bacterial treatments. The first five treatments (above the second horizontal line) were designed to compare the impacts of individual protists or the protist mixture on plant rhizosphere communities and plant growth, while the remaining three treatments aimed to examine the impacts of different bacterial inocula or the presence of the plant. Numbers of planted replicates are listed under each circled number. Six plants failed to grow, and 11 16S rhizosphere libraries had fewer than 2,000 reads, so these samples were removed from the study.

To study the fate of protist-associated bacteria in the maize rhizosphere, we inoculated maize with the eight protist cultures singly and in mixture and co-inoculated these with a bulk soil bacterial community cultured from our Lockwood Farm field site (BSC1, Fig. 2). To determine the effects of different bacterial inocula originating from different soil samples, additional plants were inoculated with bulk soil communities from Griswold (BSC2) or a rhizosphere soil community from the Lockwood site (RSC, Fig. 2) and co-inoculated with the eight-protist mixture. Maize rhizosphere bacterial communities were profiled by amplicon sequencing of the 16S rRNA genes 6 weeks after inoculation.

#### *Colpoda* spp., but not other inoculated protists, were consistently abundant in maize rhizosphere after 6 weeks

ASVs corresponding to four of the eight inoculated protists were detected in the rhizosphere samples after 6 weeks ( Table S3). All were found to establish in the maize rhizosphere 3 weeks after inoculation in our previous growth chamber study: *Cercomonas* sp. (UC19), *Ochromonas* sp. (UC48), *Thaumatomonas* sp. (UC70), and *Colpoda* sp. (UC22) (19). In this study, the former three protists were detected inconsistently at 6 weeks and at low read frequency (<0.01% of eukaryotic reads). In contrast, the *Colpoda* isolate ASVs consistently dominated protist sequence in inoculated pots, comprising an average of 20% of the eukaryotic reads (Table S3). Maize protist communities are highly temporally dynamic (21), and this result suggests that *Colpoda* sp. is uniquely persistent as a plant inoculant. Alternatively, the differences in detection could reflect differences in the encystment behavior of protists as DNA yields are lower from encysted protists than active protists (22).

Bacterial communities in the rhizosphere samples were highly variable within each of the three bacterial treatments, and bacterial inoculation did not significantly affect rhizosphere diversity or plant biomass (Fig. S1). The lack of effect of bacterial inoculation suggested that bacteria from non-inoculum sources, such as the open greenhouse environment or maize seed, primarily determined the rhizosphere bacterial community composition.

#### The maize rhizosphere consistently recruited specific protist-associated bacteria

**Fig 3:**
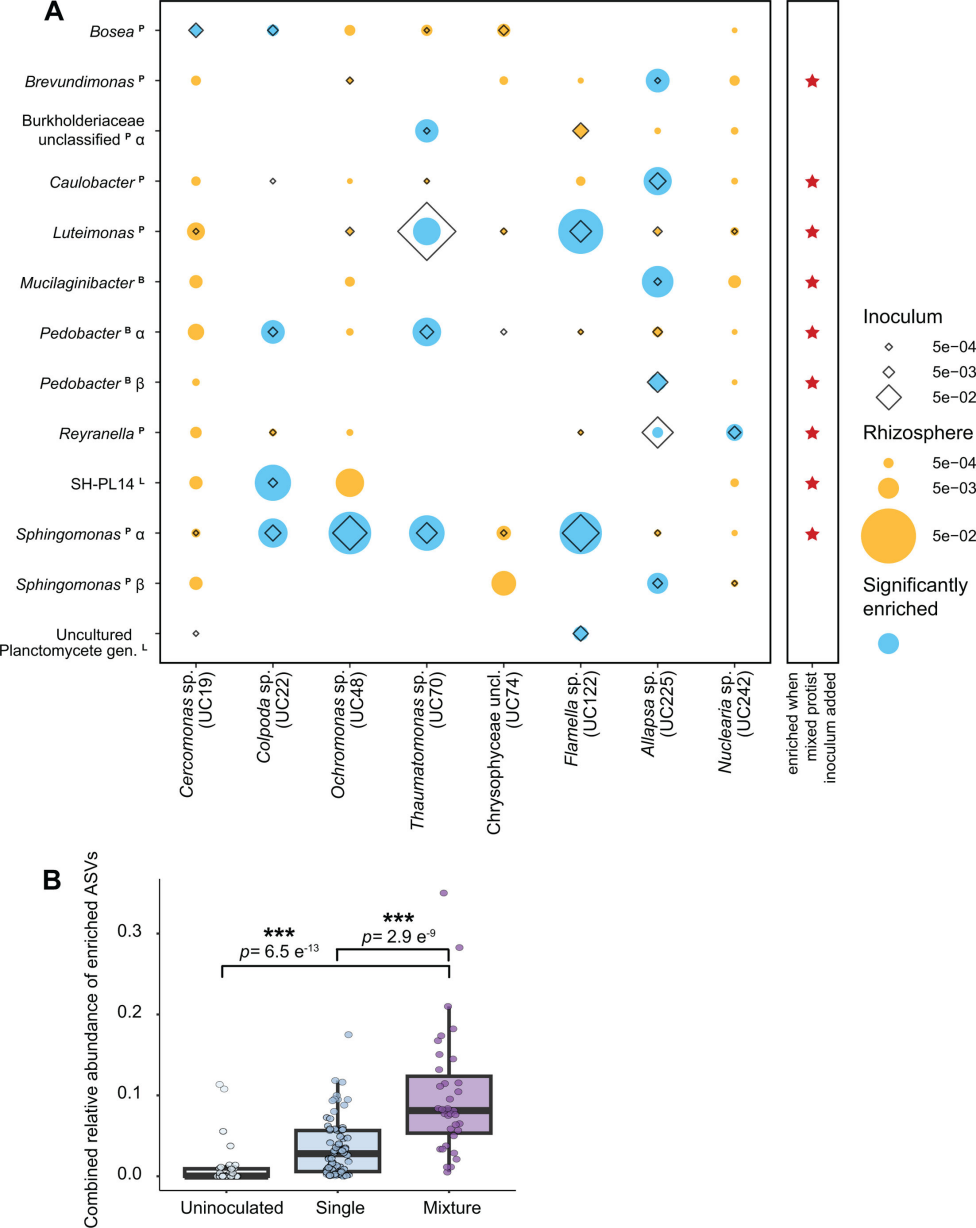
Enrichment of bacterial ASVs in the maize rhizosphere 6 weeks after protist inoculation onto germinated seeds. (**A**) Enrichment by single protists. Diameter of diamonds and dots indicates the mean relative abundance of ASVs in the protist culture microbiome (*n* = 1) and the rhizosphere samples (*n* = 9 or 10), respectively. Blue indicates statistical enrichment in the protist treatment relative to protist-uninoculated controls, as determined by DeSeq2. Red stars indicate that the bacteria were enriched when the eight protists were inoculated as a consortium. (**B**) The proportion of rhizosphere reads representing protist-enriched ASVs after plants were inoculated with single-protist cultures or mixed protists, or uninoculated. Asterisks indicate significant differences from the mixed protist treatment using Dunnett’s multiple comparison test. ***P < 0.0005.

Thirteen bacterial ASVs were significantly enriched in the rhizosphere after inoculation with individual protist cultures, in comparison with the protist-free treatments. These were enriched even when the corresponding protists were not detected (Fig. 3A; Fig. S2). Bacterial taxa enriched in the rhizosphere typically comprised greater than 0.5% of the corresponding protist-associated bacterial community (Fig. 3A; Table S4), and nine of the enriched ASVs represented dominant culture genera identified in Fig. 1. A median 8.4% of the reads in each protist microbiome corresponded to ASVs enriched in the rhizosphere (0.8%–44.0%). Over 17% of the bacteria associated with *Thaumatomonas*, *Flamella*, and *Allapsa* cultures were enriched in the rhizosphere. Some ASVs that were relatively rare among protist-associated bacteria were also enriched in the rhizosphere relative to the protist-free control, including *Brevundimonas* and an unclassified Burkholderiaceae. Many highly abundant protist-associated bacteria, such as *Variovorax* (Fig. 1), were not enriched compared to protist uninoculated plants, indicating that high abundance, in association with the protist, was not predictive of rhizosphere enrichment.

Eight (61%) of the enriched bacterial ASVs were enriched by only one protist treatment. In contrast, a *Sphingomonas* ASV (*Sphingomonas-*α) was broadly enriched after inoculation with four of the eight protist cultures and comprised up to 14.3% of the rhizosphere microbiome after inoculation. *Sphingomonas-*α was detected among all protist-associated bacterial communities except that of *Nuclearia*. Collectively, these results demonstrate that a substantial proportion of protist-associated bacteria may colonize the rhizosphere, even after prolonged laboratory cultivation of protist isolates.

#### Inoculating with multiple protists increased the abundance of protist-associated bacteria in the rhizosphere

Nine of the 13 protist-enriched bacterial ASVs were also enriched in the rhizosphere after inoculation with an eight-protist mixture (Fig. 3A), indicating that inoculating diverse protist cultures has an additive effect in enriching protist-associated bacteria. The abundance of eight protist-associated bacterial taxa did not significantly differ whether single or mixed protists were inoculated (Fig. S2), even though the mixture contained a one-eighth dilution of each culture. However, the more diverse inoculum had a negative effect on the relative abundances of *Sphingomonas*-α, *Mucilaginibacter*, unclassified Burkholderiaceae sp., and two uncultured Planctomycetales sp., which were significantly lower in the mixed than single treatments (Fig. S2). Mixed protist inoculation resulted in an increased overall abundance of protist-associated bacteria in the rhizosphere (Fig. 3B). Thirteen protist-enriched bacterial ASVs comprised a median 9.9% of the rhizosphere bacterial microbiome after protist mixture inoculation but only comprised 3.5% of the microbiome after inoculation with single protists.

#### Treatment with some protist cultures benefited root growth when inoculated individually but not in mixture

**Fig 4:**
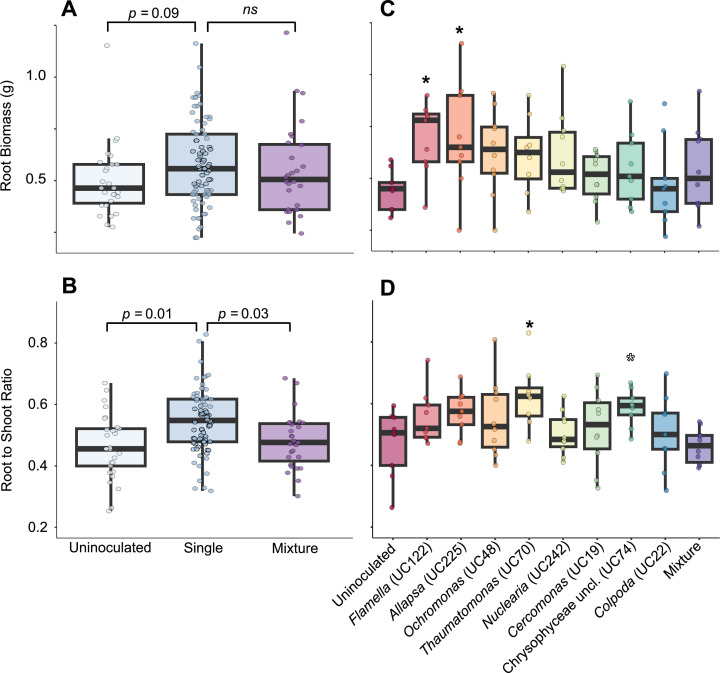
Biomass traits of maize plants inoculated with single protists and an eight-member consortium in a greenhouse study. (**A**) Root biomass and (**B**) root-shoot mass ratio 6 weeks after inoculation with zero (*n* = 29), one (*n* = 78), or a mixture of eight protists (*n* = 29) onto germinated maize seeds. (**C and D**) Root biomass (**C**) and root-shoot mass ratio (**D**) after individual protist inoculation in the presence of protist-free soil bacteria (*n* = 10 for all treatments except UC22 and UC74, which had one plant fail to grow). *P* values and asterisks indicate differences from the single-protist reference group (**A and B**) or the uninoculated reference group (C and D) using Dunnett’s multiple comparison test (filled asterisk = *P* < 0.05; open asterisk = *P* < 0.1).

We previously observed that protist-associated bacteria were necessary for a biocontrol effect of protists, resulting in increased biomass (19). Therefore, we hypothesized that inoculation with mixed protist cultures would have a greater plant biomass effect than individual cultures due to the greater number and diversity of protist-associated bacteria enriched on the root. Among all data combined, plants inoculated with single protists had a greater root:shoot biomass ratio than uninoculated plants and showed a non-significant trend toward greater root biomass, but plants inoculated with the protist mixture did not (Fig. 4A and B). Among single-protist treatments, *Thaumatomonas* culture-treated plants had increased root:shoot biomass ratios compared to the negative control, and *Flamella* and *Allapsa* treatments had increased root biomass (Fig. 4C and D). *Flamella* and *Allapsa* had the greatest bacterial richness among the protist cultures (Table S1) and, with *Thaumatomonas*, had the greatest abundance of rhizosphere-enriched bacteria (Table S4), suggesting that bacterial richness or dominance of rhizosphere bacteria in the protist microbiome could be associated with greater biomass impact. However, no significant biomass difference was observed in plants treated with the protist mixture (Fig. 4C and D). Hence, the increased relative abundance of some protist-associated bacteria observed after inoculation with the protist mixture did not translate to an increased plant biomass effect and instead may have precluded the growth effects associated with *Thaumatomonas*, *Flamella*, and *Allapsa* treatments. Given the complex interactions among protists and bacteria, the taxonomic composition of enriched bacteria, rather than the overall richness or proportion of rhizosphere colonizers, may be more important for plant growth.

#### Protist culture inoculation was less impactful in the presence of a rhizosphere bacterial community

**Fig 5:**
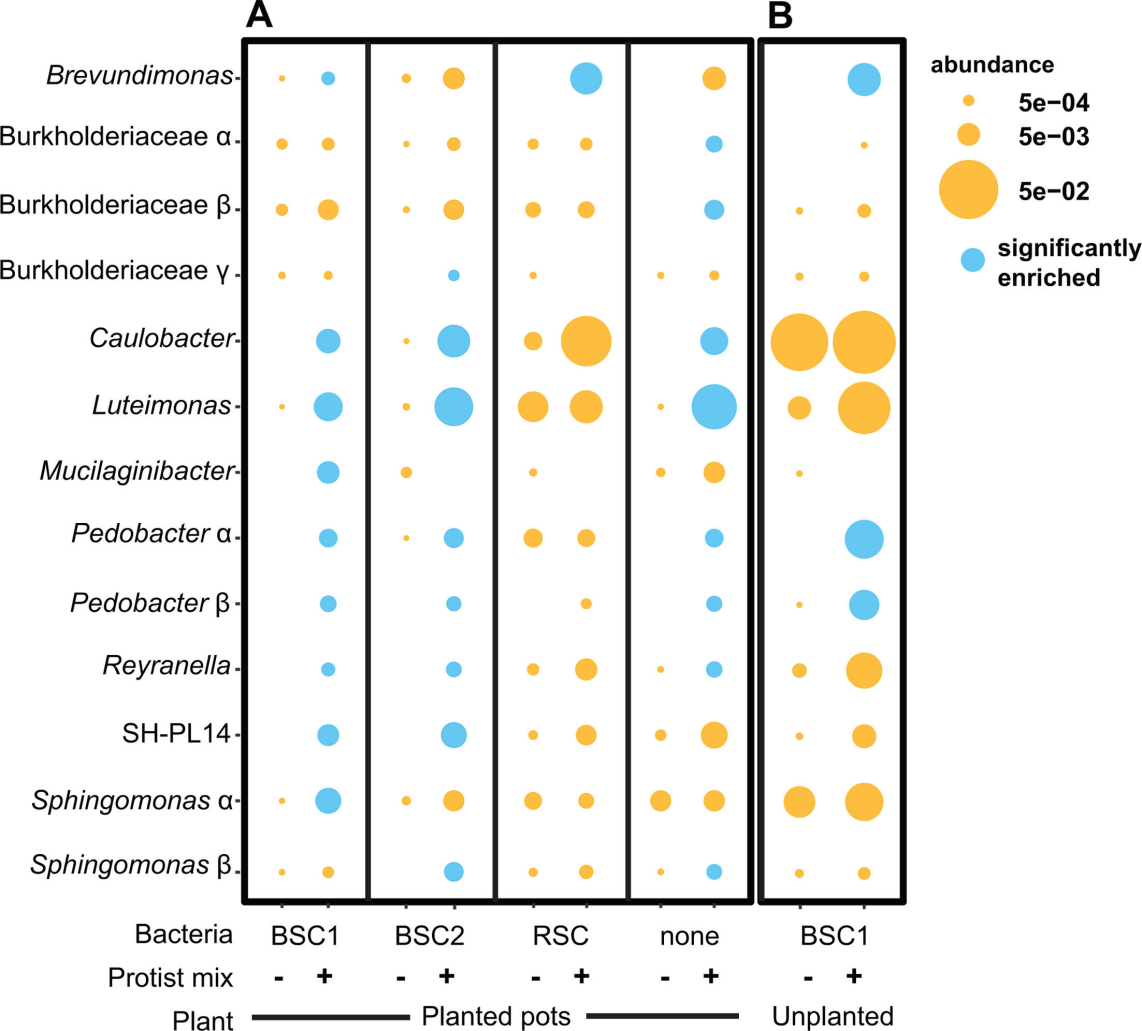
Effect of bacterial source (**A**) and presence of maize plants (**B**) on protist enrichment of soil bacteria in the rhizosphere. Enrichment of bacteria is shown when protists are added (protist mix “+”) compared to when protists are absent (protist mix “−“). (**A**) The eight-protist consortium was co-inoculated with bulk soil bacteria from Lockwood Farm (BSC1), Griswold Farm (BSC2), maize rhizosphere soil bacteria from Lockwood Farm (RSC), or no bacteria. (**B**) Enrichment of bacterial abundance after the eight-protist consortium was inoculated into the center of unplanted pots. Dot diameter indicates the mean relative abundance of the ASV (*n* = 9 or 10). Blue indicates statistical enrichment in the protist mixture treatment relative to protist-uninoculated controls within each column.

To determine whether the rhizosphere colonization of protist-associated bacteria was influenced by the surrounding bacterial community, we co-inoculated protist mixtures with bacterial communities cultured from three soil sources. Bacterial enrichment patterns following protist inoculation were similar among plants treated with bulk soil communities or no bacterial treatments; seven ASVs were enriched in multiple treatments (Fig. 5A). However, when protists were co-inoculated with rhizosphere bacterial community obtained from mature maize plants (RSC), only one ASV was enriched. We hypothesized that the rhizosphere soil community was a source of some protist-associated bacteria, and therefore, they were not enriched by the protist inoculations. By comparing RSC-inoculated plants with controls, we confirmed that inoculation with RSC was sufficient to increase the rhizosphere abundance of four protist-associated ASVs in the rhizosphere (Fig. S3A). RSC-treated plants also had a higher combined abundance of protist-enriched taxa than bulk soil communities, and protist inoculation of RSC-treated plants did not increase the total abundance of protist-associated bacteria or their abundance as observed in other treatments (Fig. S3B). This supports our previous finding that protist inoculation may substitute for rhizosphere soil in enriching certain bacterial taxa in the environment (19).

We asked whether the rhizosphere environment was required for enrichment of protist-associated bacteria or if protists alone were sufficient to enrich their abundance in soil. In unplanted pots, only *Brevundimonas* and *Pedobacter* ASVs showed increased abundance after protist inoculation, while others were enriched only in the presence of the plant (Fig. 5B). This result indicates that some protist-associated bacteria require the rhizosphere environment to become enriched in the soil. Unclassified Burkholderiaceae α was not consistently detected in unplanted pots (Fig. 5B; Fig. S3C), indicating that this taxon may obligately require either plant or protist hosts for environmental persistence.

### Seventeen additional bacterial ASVs were abundant in both rhizosphere soil and protist cultures

We detected diverse rhizosphere bacterial communities on both inoculated and untreated plants (Fig. S1, Table S5), which could mask potential enrichment of some bacteria after protist inoculation. To determine whether other bacterial taxa colonized both rhizosphere and protist microbiomes, we identified ASVs in high rank abundance in both protist culture (top 20) and rhizosphere samples of the plants inoculated with the corresponding single protist (top 100). Thirty ASVs met these criteria, of which 17 had not been enriched by protist inoculation (Table 1; Table S6 to S8). Shared abundant ASVs included representatives of dominant genera that were abundant in protist cultures, including *Azospirillum*, *Pseudomonas*, and *Bradyrhizobium*. Notably, a *Variovorax* ASV that was highly abundant in most protist microbiomes was also usually among the top 20 most abundant taxa on maize (Table S6). Together, ASVs that were enriched or abundant on plants comprised a median 46.8% of the reads in the protist culture microbiomes (Table S4, range 6.6%–67.8%). This shows that several rhizosphere protist cultures were dominated by candidate rhizosphere-colonizing bacteria, including a wider range of taxa than was enriched by protist inoculation.

**TABLE 1:** Protist culture microbiome bacterial ASVs that were enriched or abundant in the rhizosphere after protist inoculation

ASV^*a*^	Genus	Protist culture ID^*b*^	Enriched in rhizosphere^*c*^	Abundant in rhizosphere^*d*^
1	*Azospirillum*	UC19, UC48, UC74, and UC122		X^*e*^
2	*Bosea*	UC74		X
3	*Bosea*	UC19, UC22, and UC74	X	X
4	*Bradyrhizobium*	UC19, UC22, UC48, UC70, UC74, and UC122		X
5	*Brevundimonas*	None	X	
6	*Brevundimonas*	UC242		X
7	Burholderiaceae, unclassified	UC22		X
8	Burkholderia, unclassified	UC74		X
9	Burkholderiaceae, unclassified	UC122	X	X
10	*Caulobacter*	UC225 and UC242	X	X
11	*Caulobacter*	UC19 and UC225		X
12	*Cupriavidus*	UC225		X
13	Enterobacteriaceae, unclassified	UC48		X
14	*Luteimonas*	UC70 and UC122	X	X
15	*Mucilaginibacter*	UC19 and UC22		X
16	*Mucilaginibacter*	None	X	
17	*Paenarthrobacter*	UC242		X
18	*Pedobacter*-α	UC22 and UC70	X	X
19	*Pedobacter*-β	UC225	X	X
20	*Pseudomonas*	UC19, UC48, and UC74		X
21	*Pseudoxanthomonas*	UC122 and UC19		X
22	*Reyranella*	UC225 and UC242	X	X
23	*Reyranella*	None		X
24	*Rhodococcus*	UC19, UC22, UC48, UC74, UC122, and UC242		X
25	*Sphingomonas*-α	UC22, UC48, UC70, and UC122	X	X
26	*Sphingomonas*-β	None	X	
27	Planctomycetales, unclassified	UC122	X	X
28	Xanthobacteraceae, unclassified	UC48, UC70, UC122, and UC225		X
29	Uncultured gen. SH-PL14	None	X	
30	*Variovorax*	UC19, UC22, UC48, UC70, UC74, UC122, and UC242		X

^*a*^ ASV identifiers are found in Table S5.

^*b*^ Culture codes represent the following protists: UC19, *Cercomonas*; UC22, *Colpoda*; UC48, *Ochromonas*; UC70, *Thaumatomonas*; UC74, *Chrysophyceae*, unclassified; UC122; *Flamella*; UC225, *Allapsa*; UC242, *Nuclearia*.

^*c*^ Significantly enriched in maize rhizosphere 42 days after single-protist inoculation in greenhouse, relative to uninoculated plants.

^*d*^ Among the 100 most abundant bacterial ASVs in the rhizosphere 42 days after protist inoculation, regardless of enrichment.

^*e*^ X denotes that the ASV is present in the corresponding category.

### Rhizosphere-enriched bacterial genera may promote or suppress protist growth

**Fig 6:**
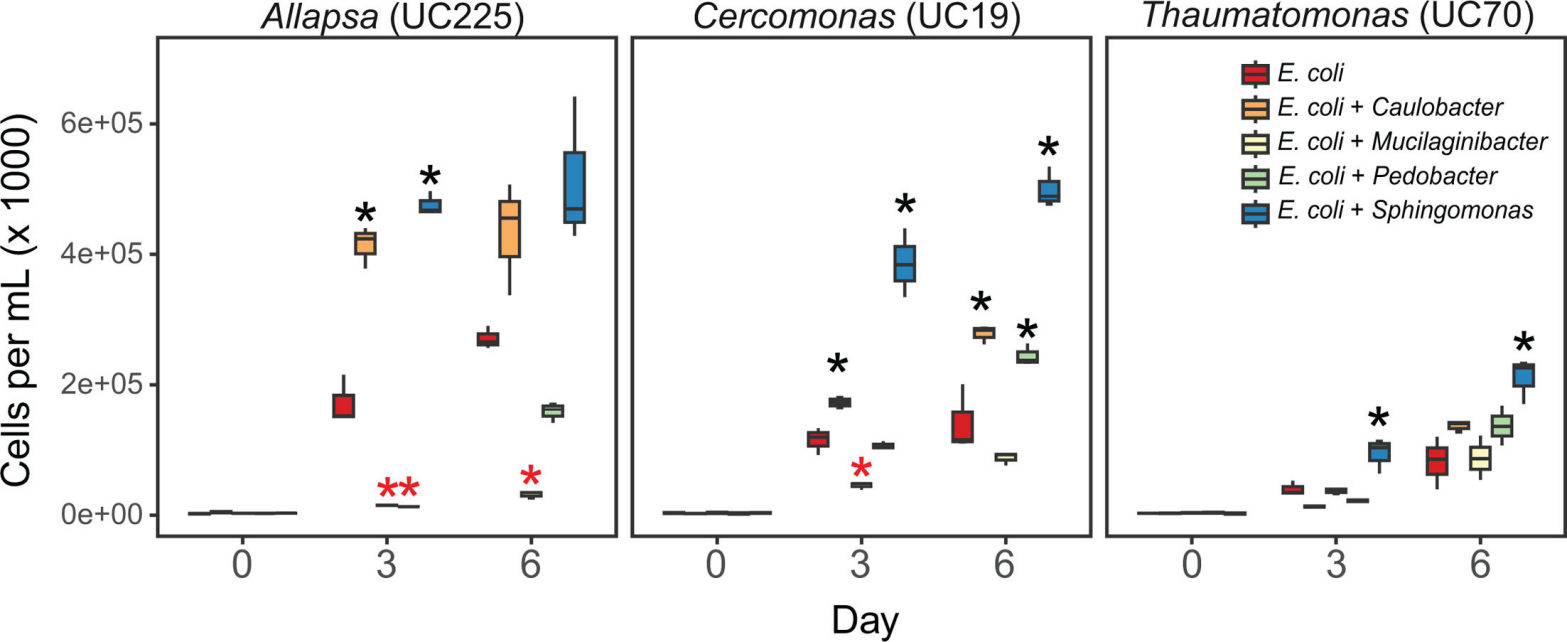
Cell counts of three protist taxa (*Allapsa*, *Cercomonas*, and *Thaumatomonas*) after being fed with heat-killed *E. coli* alone, or with heat-killed *E. coli*, in addition to one of four bacterial cultures isolated from protist cultures (*n* = 3 for each treatment). Cells were counted at 0, 3, and 6 days after the cells were initially fed. Black and red asterisks indicate increased and decreased protist counts after adding the bacterial isolates: black asterisks indicate that there were significantly more protists with the bacterial treatment than with heat-killed *E. coli* alone, while red asterisks indicate that there were significantly fewer protists (*P* < 0.05).

We lastly wondered why many rhizosphere-colonizing bacteria were able to persist for long periods in association with diverse types of protists. We hypothesized that the protist-associated bacteria are not a food source for the protists or, alternatively, that they could provide a benefit to the protists. To test this, we isolated four bacteria from the protist cultures having 16S rRNA gene similarity to rhizosphere-enriched ASVs *Sphingomonas*-α (100% similarity), *Pedobacter*-β (98.4%), *Caulobacter* (98.0%), and *Mucilaginibacter* (96%). These genera were all enriched in the rhizosphere after inoculation with *Allapsa* (Fig. 3), which had a positive effect on root biomass. We measured the effects of these bacterial isolates on antibiotic-treated cultures of *Allapsa*, as well as two other isolates from phylum Cercozoa, *Cercomonas*, and *Thaumatomonas*, in which the four bacterial genera were detected (Fig. 1) but variably enriched (Fig. 3). All protists grew when cultures were supplemented with *Caulobacter* or *Sphingomonas* but not when *Pedobacter*, *Mucilaginibacter*, or no bacteria were provided (Fig. S4); this indicates that *Pedobacter* and *Mucilaginibacter* isolates were not a source of prey (15). To determine whether the bacterial isolates suppress growth, we measured the effects of the bacteria on protists supplemented with an excess of inert prey (heat-killed *Escherichia coli*). At 3 days, *Sphingomonas* increased the growth of all three protists, increasing the density of the cultures by 2.4- to 3.3-fold compared to *E. coli* alone. *Caulobacter* sp. had a strong positive effect on the growth of *Allapsa* (2.4×, *P* < 0.001), a smaller effect on *Cercomonas* (1.5×, *P* = 0.066), and no effect on *Thaumatomonas* (Fig. 6). Conversely, *Pedobacter* and *Mucilaginibacter* were strongly antagonistic to *Allapsa* growth*,* had differing positive and negative effects on *Cercomonas*, and had no significant effect on *Thaumatomonas*. Taken together, the results show that protist-associated rhizosphere bacteria use differing strategies to persist under predation pressure, supporting or inhibiting protist growth in broad or isolate-specific ways.

## DISCUSSION

By surveying the bacteria associated with diverse protist cultures and tracking their fates in the rhizosphere, this study demonstrates that a wide diversity of rhizosphere microeukaryotes is associated with many plant-colonizing bacterial taxa. Prior to these findings, green algae were the only unicellular eukaryotes known to recruit bacterial communities with strong functional parallels to those of plants (16), making the algal microbiome a proposed source of plant-beneficial bacteria (23). Our study shows that diverse predatory eukaryotes outside the Chloroplastida also associate with rhizosphere-colonizing bacteria in culture. Rhizosphere bacteria persisted for approximately 2 years in active cultures of protists from Stramenopila, Alveolata, and Rhizaria (SAR), Excavata, and Amoebozoa supergroups and maintained the capacity to colonize roots in a complex microbial environment. The findings support the hypothesis that, in addition to their roles imparting selective and behavioral advantages to rhizosphere bacteria (24), predatory soil protists could recruit or promote a bacterial microbiome that contributes to rhizosphere establishment. Understanding how these bacteria interact with protists could help identify defense and signaling mechanisms important to bacterial survival and may ultimately help enable the design of improved microbial consortia to benefit agriculture.

Profiling the protist-associated bacteria revealed a broad taxonomic overlap among protists independently isolated from different plants and field sites, indicating the broad prevalence of Burkholderiales and Hyphomicrobiales. They also included groups previously documented to survive protist internalization: members of *Sphingomonas*, *Burkholderia*, *Pseudomonas*, *Pseudoxanthomonas*, *Pedobacter*, *Mesorhizobium*, and *Acidovorax* have been identified in protist cells (25–28). *Variovorax* spp. are common epibionts and endosymbionts of protists (28, 29), and *Bosea* and *Reyranella* spp. are free-living protist mutualists that can be isolated using a strategy of protist co-culture (30, 31). While these studies support protist selection of the taxa identified in this study, it is also important to note that protist microbiome composition is likely shaped in part by the local soil microbiome, stochastic events during isolation, culture conditions, and bacteria-bacteria interactions. The protist-associated communities identified here likely only represent a portion of true protist interactors in soil. More work is needed to confirm which features of protist microbiomes represent replicable selection patterns and true symbioses.

Our previous study found that inoculation with an 18-protist mixture in a closed growth chamber did not significantly change rhizosphere microbiome structure in the presence of soil bacteria but enriched the abundance of bacteria from a few specific genera relative to the bacteria. Here we used a smaller consortium to confirm that observation and demonstrate that each protist culture made a few distinct contributions to the rhizosphere community. We also demonstrated that these changes could be observed after 6 weeks, when many of the corresponding protists could no longer be detected. Some enriched genera identified here were previously correlated with protist abundance in plot studies, indicating that protist-bacterial network connections might reflect microbiome associations that are robust in the field. For example, *Luteimonas*, *Brevundimonas*, *Pseudomonas*, and *Pseudoxanthomonas* were positively correlated with a *Colpoda* sp. in tomato rhizosphere (32). In this study, the ASV *Sphingomonas*-α was enriched to high levels by the widest spectrum of protists; we previously identified the same ASV as the largest protist network hub on several field-grown solanaceous crops (33). *Sphingomonas* abundance was previously linked to increased plant growth following *Cercomonas* inoculation on cucumber seedlings, and *Cercomonas* triggered biofilm formation of the bacteria *in vitro* (34). Our own observations that *Sphingomonas*-α is correlated with protists in the field, found in microbiomes of diverse protist cultures, enriched in the rhizosphere after protist inoculation, and broadly promotes protist growth *in vitro* suggest that *Sphingomonas* could be a partner in widespread synergistic associations with protists and plants. Enriched bacteria included two ASVs associated with uncultured *Planctomyces* species, indicating that protist inoculation could be a means to allow rhizosphere introduction of some unculturable bacteria to the plant.

The effects of protist diversity on microbial communities are variable and can shift dynamically under different environmental and biotic stress conditions (35, 36). We observed that inoculation with a mixture of protist cultures enriched a greater number and abundance of rhizosphere bacteria compared with single-protist inoculation, but only a few single protists affected biomass traits. The increase in inoculum diversity may have affected the activity and distribution of bacteria through intermicrobial interactions, resulting in decreased abundance of potentially beneficial bacteria such as *Sphingomonas* and *Mucilaginibacter* (Fig. S1). Even when reducing protist diversity, changing community composition greatly shaped protist effects on the maize rhizosphere bacterial microbiome. Of 21 protist-associated bacterial genera found to colonize plants in this study, only nine were enriched by an 18-protist consortium in our previous study (19) (Table S8). The 18-protist consortium enriched the abundance of some taxa not enriched in this study, particularly the genus *Duganella*. We have recently determined that *Duganella* is found specifically associated with an *Ochrophyta* sp. isolate that was excluded from this study (UC29; R. R. Patel et al., unpublished data). As we have observed in this study, inclusion of a protist isolate hosting even a few rhizosphere-colonizing bacteria can have a significant impact on the microbiome. The observation of parallels among plant and protist microbiomes raises many questions: what is the mechanism and nature of protist interactions with its microbiome? Do protists provide the bacteria a fitness or other plant colonization advantage in the rhizosphere, or induce the bacteria to benefit the plant? The interactions identified here provide a starting point for understanding the ecology and agricultural significance of protist-associated bacteria on plants.

## MATERIALS AND METHODS

### Culture preparation

We selected protist cultures from a collection isolated from maize roots in 2020 (20). Protist cultures were maintained in PAGE amoebal saline (i.e., PAGE solution; 0.12 g of NaCl, 0.004 g of MgSO_4_·H_2_O, 0.004 g of CaCl_2_, 0.142 g of Na_2_HPO_4_, and 0.136 g of KH_2_PO_4_ in 1 L of dH_2_O) amended with heat-killed *E. coli* as previously described (19). Protists had been maintained with monthly passaging for 2 years at the time of DNA extraction and inoculation onto plants. To prepare cultures for plant inoculation, protists were transferred to sterile PAGE solution and fed with heat-killed *E. coli* weekly for 1 month, grown just until encystment, centrifuged for 30 min at 2,000 × *g*, resuspended in 4 mL PAGE solution, and pelleted again for 15 min at 2,000 × *g* before resuspension in 500 μL PAGE solution. Protist concentration was determined by counting under a microscope, then protists were diluted in PAGE solution to a concentration of 1,000 cells/mL. Cultures were mixed in equal proportion and then diluted to 1,000 cells/mL to create the eight-protist mixture.

To generate protist-free soil bacteria, we collected 10 cm-deep soil cores from five random locations in maize fields from Lockwood Farm (Hamden, CT) and Griswold, CT. We suspended 10 g of soil in 100 mL of sterile phosphate-buffered saline (PBS; pH 7.4; 8 g of NaCl, 1.44 g of Na_2_HPO_4_, 0.2 g of KCl, and 0.24 g of KH_2_PO_4_ in 1 L of dH_2_O). Rhizosphere soil was collected from five plants of maize (*Zea mays* L.) inbred line B73 (stage V5) collected from the Lockwood field on the same day as the bulk soil. Roots were shaken to remove excess soil, and five roots per plant were agitated in sterile PBS to collect rhizosphere soil. Soil samples were plated in serial dilutions on both tryptic soya agar and Reasoner’s 2 agar plates (0.25 g of casein digest peptone, 0.25 g of peptic digest of animal tissue, 0.5 g of MgSO_4_·7H_2_O, 0.3 g of C_3_H_3_NaO_3_, 0.5 g of casein acid hydrolysate, 0.3 g of K_2_HPO_4_, 0.5 g yeast extract, 0.5 g soluble starch, 0.5 g glucose, and 15 g agar in 1 L of dH_2_O), which were incubated at 28°C for 72 h. After incubation, the bacterial colonies from each plate were scraped and collected in PBS containing 20% glycerol and mixed by vortexing. Suspensions were stored at −80°C until use. One aliquot was used for colony enumeration. Bacterial inoculum for plant experiments was then prepared by thawing the tubes on ice and resuspending to 10^6^ cells/mL in sterile PBS.

### Plant inoculation and growth

We prepared a soil mixture containing 53% sand, 37% vermiculite, and 10% field soil from Lockwood Farm, which is the same field from which all but two of the protists were originally isolated. The mixture was wetted and autoclaved twice in 12 cm pots. Seeds of B73 maize were surface sterilized in 3% NaOCL for 1 min, then washed 10 times in sterilized dH_2_O. The seeds were germinated on sterile filter paper in the dark at 28°C for 2 days. In all but 20 pots, germinated seeds were placed into the middle of the pots at a depth of 4 cm and inoculated with 1 mL of protist inoculum (at a concentration of 10^3^ cells/mL) or sterile PAGE solution, then with 1 mL of bacteria (10^6^ cells/mL) or sterile phosphate buffered saline (pH 7.4), depending on the experimental treatment. Germinated seeds were inoculated by slowly pipetting the solution directly onto the emerging radicle before covering with the soil mixture. No germinated seeds were placed in the remaining 20 pots; instead, soil was removed to 4 cm in the center of the pot, and the soil in this location was inoculated with 1 mL of bulk soil bacteria and 1 mL of mixed protist inoculum (10 pots), or 1 mL of bulk soil bacteria alone (10 pots) before being covered by soil. Pots were arranged in a randomized block design and maintained in the greenhouse for 6 weeks during June and July 2021 (16 h days, temperatures 25°C–30°C). Plants were watered with autoclaved distilled water when pot weights decreased. At collection, roots were shaken vigorously to remove excess soil and then placed in plastic bags containing 35 mL PBS. Roots were agitated for 30 seconds to remove attached soil, after which the rhizosphere suspensions were transferred to 50 mL tubes and stored at −80°C until DNA extraction. Root and shoot biomass was recorded after drying for 1 week at 50°C. For the plant-free replicates, soil was removed to a depth of 4 cm, and ~1 g of soil was removed and transferred to a 50 mL tube containing 35 mL PBS.

### 16S and 18S rRNA gene library preparation

DNA extractions, PCR amplifications, and sequencing were conducted following previously published protocols (19) (summarized in Table S9). The Illumina sequences can be found in National Center for Biotechnology Information (NCBI) GenBank under BioSample PRJNA950058.

### Read assembly and classification

We assembled the reads using mothur v.1.44.0 (37). 16S contigs >300 bp and 18S contigs <90 or >250 bp were removed, as were contigs containing ambiguous bases or homopolymers greater than eight bases. Chimeras were removed in mothur using VSEARCH (38). 16S ASVs were classified using the SILVA v.132 database, while 18S ASVs were classified using the Protist Ribosomal Reference v.4.12 database (39). Both classifications used the naïve Bayesian classifier implemented in mothur. Libraries with fewer than 2,000 reads were excluded from further analyses.

The sterilized PAGE solution sample was analyzed as a negative control. For the 16S analyses, the control run totaled 7.9% reads of the next smallest library, while for the 18S analyses, the control run totaled 7.7% reads of the next smallest library.

### Diversity and enrichment analyses

We calculated diversity metrics of inoculum samples and rhizosphere communities after subsampling to the smallest read number among inoculum samples or rhizosphere sample data sets using the phyloseq v.1.38.0 package (40) implemented in R v.4.1.2. The 16S communities were subsampled to 2,195 reads for the protist inocula, 21,547 reads for the bacterial inocula, and 2,218 reads for the rhizosphere samples, while the 18S communities were subsampled to 14,666 reads for the rhizosphere samples. Heatmap generation was performed using the MicEco v.0.9.14 package (41), after removing ASVs with fewer than five reads in the data set. Enrichment of bacterial ASVs in rhizosphere samples from plants inoculated with protists compared with plants inoculated with sterile PAGE solution (i.e., protist-free treatments) was determined using the DESeq2 v.1.34.0 package (42) implemented in R. Adjusted *P* values for DESeq2 were calculated, and false positives were estimated using the Benjamini-Hochberg method.

### Bacterial isolation and identification

Protist culture solutions were diluted onto plates of tryptic soy agar and incubated at 28°C for 3 days. Ninety-six colonies were selected and cultured at random. Bacterial genomic DNA was extracted and amplified using the primers 27F (5′ AGAGTTTGATCCTGGCTCAG 3′) and 1492R (5′ GGTTACCTTGTTACGACTT 3′) (43). Partial 16S sequences were sequenced using Sanger sequencing at the Keck DNA Sequencing Core at Yale University, New Haven, USA, and identified using Blast search in the NCBI database. Strains identified as *Mucilaginibacter*, *Caulobacter*, *Sphingomonas*, and *Pedobacter* were respectively isolated from cultures of UC70, UC70, UC122, and UC225 and chosen for the study.

### Protist growth assay

Cultures of *Allapsa* isolate UC225, *Cercomonas* isolate UC19, and *Thaumatomonas* isolate UC70 were treated with antibiotics to reduce endogenous bacteria as previously described (19). Protist cultures were grown in PAGE solution amended with heat-killed *E. coli* (O.D. 0.1) until the cells were freshly encysted. Cysts were centrifuged twice at 4,000 × *g* and resuspended in fresh PAGE solution to remove excess remaining killed *E. coli* cells, then enumerated and resuspended in PAGE solution to a concentration of 1,000 cysts/mL. Bacterial cultures were grown for 72 h in Reasoner’s 2 broth media at 28°C.

Assays were conducted in 24-well cell culture plates. Protist cells were diluted to 100 cells in 1 mL of sterile PAGE solution, while bacteria were diluted to an optical density (O.D.) of 0.07. In addition, wells were amended with heat-killed *E. coli* to an O.D. of 0.07. Three replicates each were set up of all protist-bacteria pairwise combinations, while three replicates each were set up of protists grown without added bacteria as controls. After the plates were set up, cells were visualized at ×200 magnification using a Zeiss ID02 Invertoscope inverted microscope and photographed using an Axiocam 305 color camera. Three photos were taken of each well in a triangular pattern. Cells were photographed every 24 h for the following 6 days. Cells were enumerated using ImageJ, after which the number of cells in 1 mL was calculated for each well and timepoint.

### Statistical analysis

Data were graphed using ggplot2 v.3.4.0 (44) implemented in R v.4.3.0. Statistical differences between treatments and controls were determined using analysis of variance followed by Dunnett’s multiple comparison test using the DescTools package in R. Cell count data were analyzed in R using a Dunnett test to compare with the controls.

## Data Availability

Sequences were deposited in GenBank under accession numbers PQ741793 to PQ741796.

## SUPPLEMENTAL MATERIAL

Supplemental Figures msphere.00037-25-s0001.pdf Figures S1-S4.

Supplemental Tables msphere.00037-25-s0002.xlsx Tables S1-S9.

ASM does not own the copyrights to Supplemental Material that may be linked to, or accessed through, an article. The authors have granted ASM a non-exclusive, world-wide license to publish the Supplemental Material files. Please contact the corresponding author directly for reuse.
