# Supplementary material for: Rhizosphere-colonizing bacteria persist in the protist microbiome
Source: mSphere. 2025 Apr 30;10(5):e00037-25. doi: 10.1128/msphere.00037-25 (PMC12108058; doi:10.1128/msphere.00037-25)
Supplement: Supplemental Figures — Figures S1-S4. [file msphere.00037-25-s0001.pdf]

Supplementary figure captions:

Figure S1: Effect of bacterial treatment on (**A**) the β-diversity of rhizosphere bacterial communities and (**B**) the biomass of maize plants. **A.** NMDS plot showing the clustering of 16S communities of maize rhizospheres after inoculation with bulk soil bacteria from Lockwood Farm (BSC1), Griswold Farm (BSC2), maize rhizosphere soil bacteria from Lockwood Farm (RSC), or no bacteria, and either no protists or the eight-protist consortium. Dashed lines represent 95% confidence intervals. **B.** Root biomass of maize plants six weeks after inoculation with the different bacterial treatments, with and without the protist mixture.

Figure S2: Relative abundances of the 13 enriched bacteria in maize rhizosphere samples six weeks after inoculation with zero, one, or a mixture of eight protists onto germinated maize seeds. Asterisks indicate relative abundances that were significantly greater ($p < 0.05$) compared with the mixture-inoculated plants in a Dunnett's Multiple Comparison Test.

Figure S3: Effect of rhizosphere soil on bacterial enrichment and abundance. **A.** Enrichment of five protist-associated bacterial ASVs in maize rhizosphere samples inoculated with bulk soil bacteria from Lockwood Farm (BSC1) or maize rhizosphere soil bacteria from Lockwood Farm (RSC) compared with bacteria-uninoculated controls. **B.** Relative abundance of all protist-enriched bacterial ASVs in soils with and without protist inoculation. Asterisks indicate significant difference from the corresponding uninoculated soil at $p < 0.005$ (**) and $p < 0.001$ (***). **C.** Relative abundance of the bacterial ASV "Burkholderiaceae unclassified β" in soil inoculated with the protist mixture or no protist, with or without the plant.

Figure S4: Cell counts of three protist taxa (*Allapsa*, *Cercomonas*, and *Thaumatomonas*) after being fed with no bacteria or with one of four bacterial isolates from protist cultures (n = 3 for each treatment). Cells were counted at 0, 3 and 6 days after the cells were initially fed. Black asterisks indicate significantly more protists than those fed with no bacteria ($p < 0.05$) based on a Dunnett's Multiple Comparison Test.

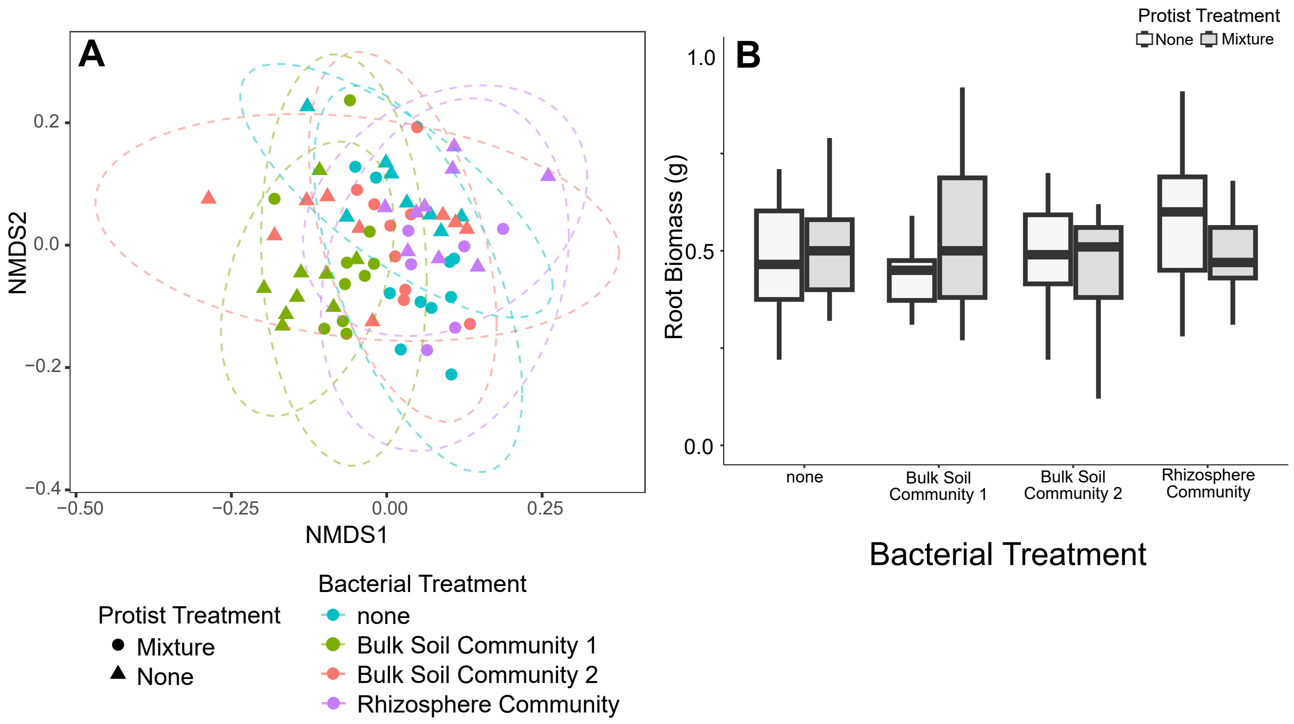

Figure S1: Effect of bacterial treatment on (A) the β-diversity of rhizosphere bacterial communities and (B) the biomass of maize plants. A. NMDS plot showing the clustering of 16S communities of maize rhizospheres after inoculation with bulk soil bacteria from Lockwood Farm (Bulk Soil Community1), Griswold Farm (Bulk Soil Community 2), maize rhizosphere soil bacteria from Lockwood Farm (Rhizosphere Community), or no bacteria (none), with or without the eight-protist consortium. Dashed lines represent 95% confidence intervals. B. Root biomass of maize plants six weeks after inoculation with the different bacterial treatments, with or without the eight-protist mixture.

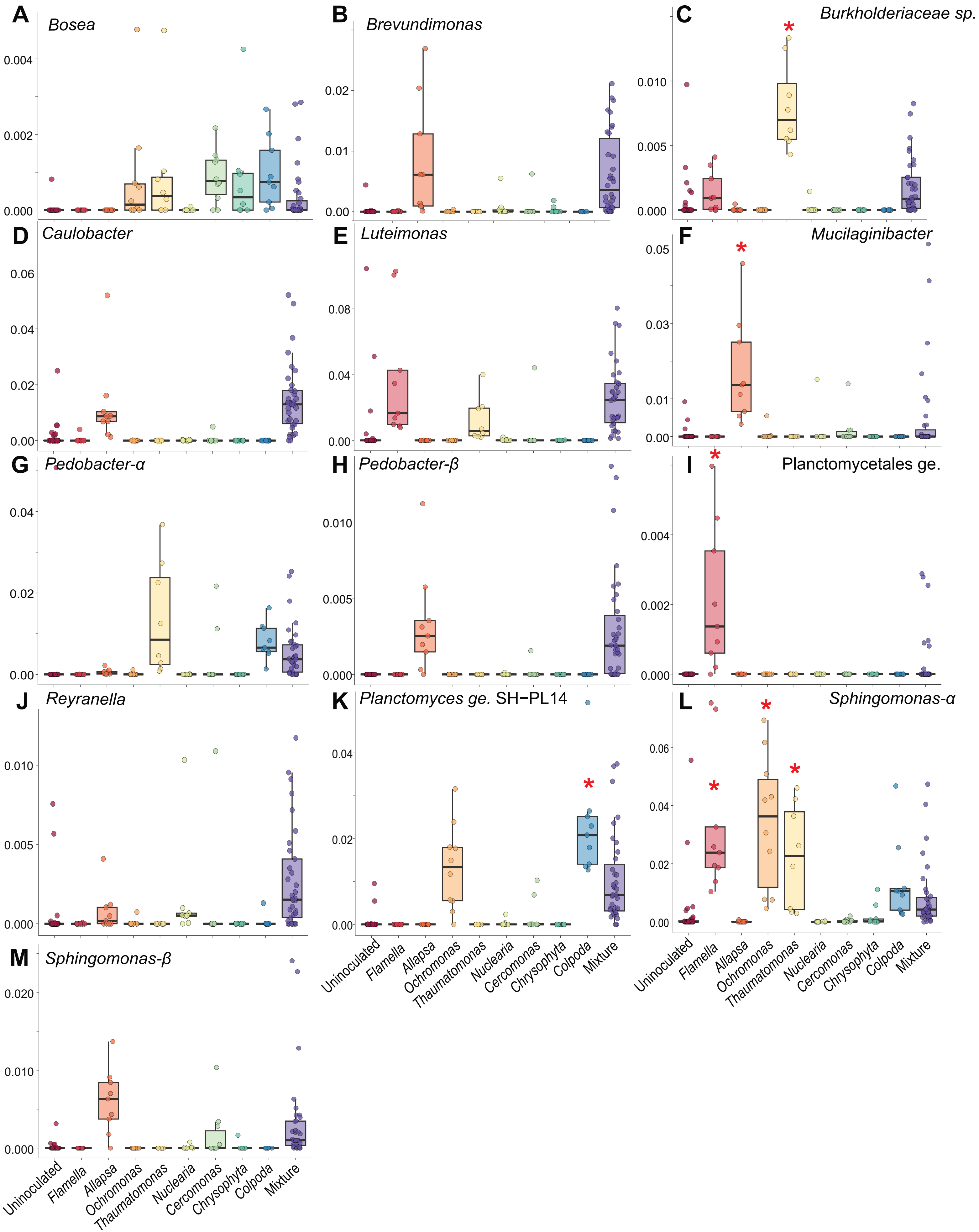

Figure S2: Relative abundances of the 13 enriched bacteria in maize rhizosphere samples six weeks after inoculation with zero, one, or a mixture of eight protists onto germinated maize seeds. Asterisks indicate that relative abundances were significantly greater ($p < 0.05$) than in the mixture-inoculated plants based on a Dunnett's Multiple Comparison Test.

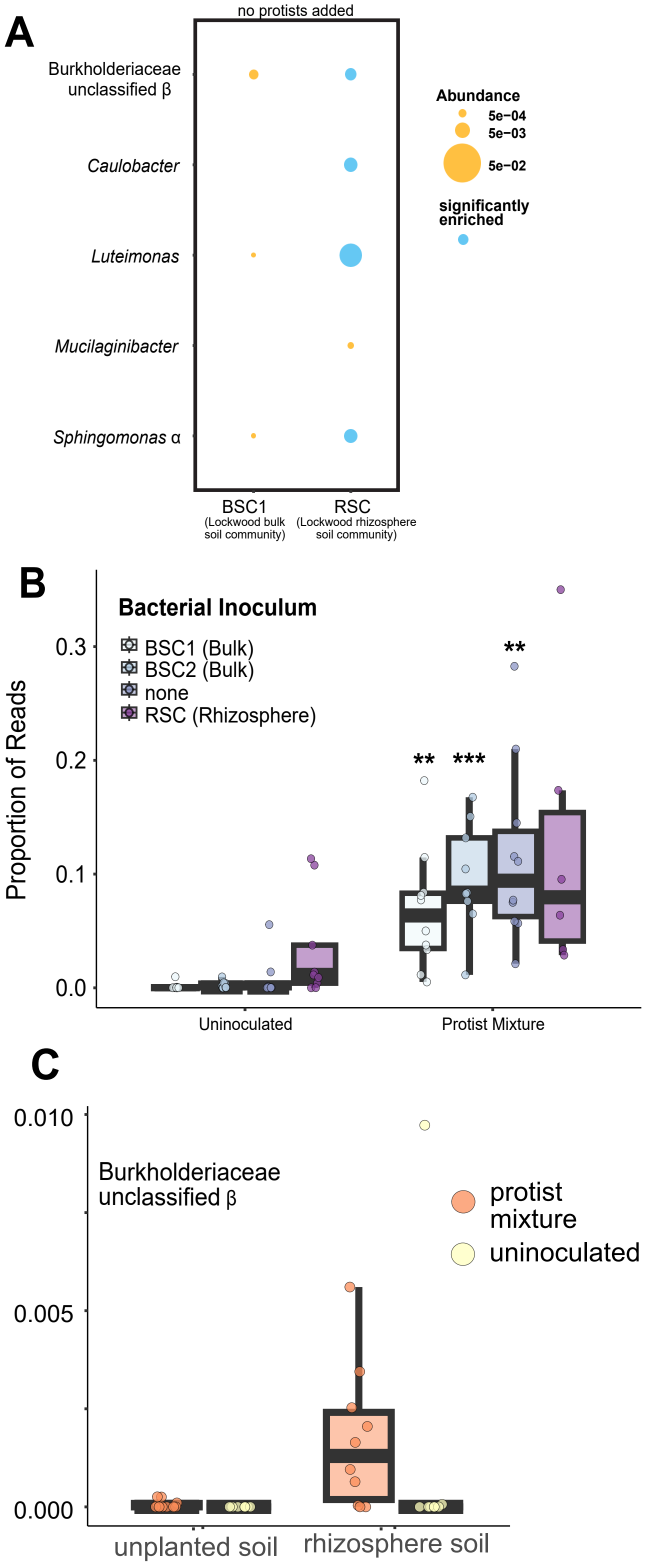

Figure S3: Effect of the rhizosphere on bacterial enrichment and abundance. A. Enrichment of five bacterial ASVs in maize rhizosphere samples from plants inoculated with bulk soil bacteria from Lockwood Farm (BSC1) or maize rhizosphere soil bacteria from Lockwood Farm (RSC) compared with uninoculated controls. Circle diameters indicate mean relative abundances of bacteria. B. Relative abundance of all protist-enriched ASVs in soils with and without protist inoculation. Asterisks indicate $p < 0.005$ (**) and $p < 0.001$ (***). C. Relative abundance of the ASV "Burkholderiaceaeunclassified β" in soil inoculated with the protist mixture or no protist, with or without the plant.

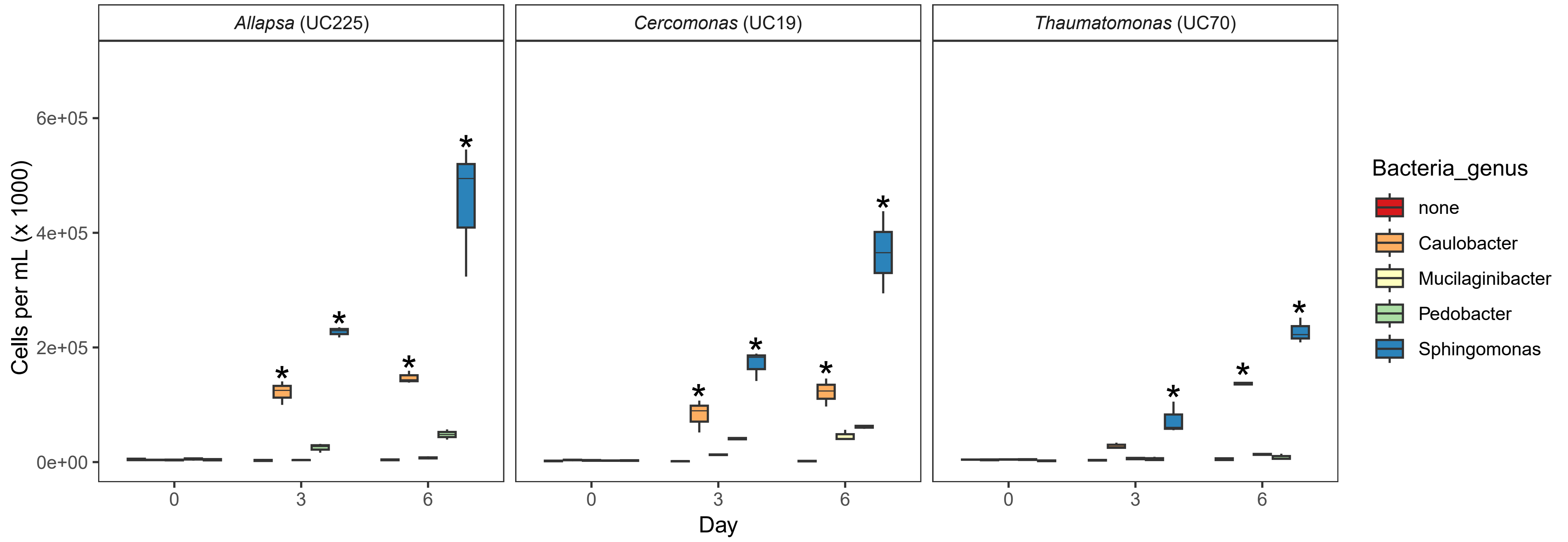

Allapsa (UC225)
Cercomonas (UC19)
Thaumatomonas (UC70)
Cells per mL (x 1000)
6e+05
4e+05
2e+05
0e+00
0
3
6
Day
Bacteria_genus
none
Caulobacter
Mucilaginibacter
Pedobacter
Sphingomonas
